# Large fluctuations at the lasing threshold of solid- and liquid-state dye lasers

**DOI:** 10.1038/srep32134

**Published:** 2016-08-25

**Authors:** Supratim Basak, Alvaro Blanco, Cefe López

**Affiliations:** 1 Instituto de Ciencia de Materiales de Madrid (ICMM) Consejo Superior de Investigaciones Científicas (CSIC), Calle Sor Juana Inés de la Cruz 3, Cantoblanco, 28049 Madrid, Spain

## Abstract

Intensity fluctuations in lasers are commonly studied above threshold in some special configurations (especially when emission is fed back into the cavity or when two lasers are coupled) and related with their chaotic behaviour. Similar fluctuating instabilities are usually observed in random lasers, which are open systems with plenty of quasi-modes whose non orthogonality enables them to exchange energy and provides the sort of loss mechanism whose interplay with pumping leads to replica symmetry breaking. The latter however, had never been observed in plain cavity lasers where disorder is absent or not intentionally added. Here we show a fluctuating lasing behaviour at the lasing threshold both in solid and liquid dye lasers. Above and below a narrow range around the threshold the spectral line-shape is well correlated with the pump energy. At the threshold such correlation disappears, and the system enters a regime where emitted laser fluctuates between narrow, intense and broad, weak peaks. The immense number of modes and the reduced resonator quality favour the coupling of modes and prepares the system so that replica symmetry breaking occurs without added disorder.

Lasers made with organic dyes in liquid solutions or embedded in solid matrices are appreciated for their high efficiency^1^. Thus random lasers (RL)^2^, a notable example combining disorder and gain media, but lacking a cavity (which hinders feedback) were demonstrated^3^ and are most often made with organic dyes. In this case the feedback is obtained from scattering off the disordered medium so no external cavity is needed. Due to their nature it is reasonable to expect some fluctuating behaviour in their emission^4^. Apart from the case where chaotic behaviour is purposefully provoked for technological applications^5^, laser fluctuations were mostly observed to occur (and fought against) above threshold, *i.e*. during laser action. For instance, fluctuations in the emitted spectra of ZnO in an organic solid matrix observed when excited with pulses longer that the chromophore lifetime were described as a lasing *instability* due to interplay between pulse length and excited state lifetime^6^. Unlike these intrinsic fluctuations, a similar system, albeit liquid, showed fluctuation attributed to the dynamic disorder within the colloid realized for each pump pulse^7^. Mujumdar *et al*. introduced the idea of mode coupling between the long lived extended modes for the chaotic behaviour of emission spectra^8^.

In any case, the lasing threshold of lasers, random or conventional, is perhaps the regime that garnered the least attention^9^,^10^. Intensity fluctuation between the emission from lasing and non-lasing modes at the threshold in cw GaAs laser were modelled using coupled van der Pol oscillators^11^. A temperature dependent study of the correlation between the fluctuations of different modes for a semiconductor laser has also been carried out^12^. The latter examples deal with very few modes and depend on direct energy exchange between *one* lasing mode and *few* neighbouring non-lasing ones. These modes are relatively far apart and respond differently to changes in gain so their noise effect is of an individual rather than collective character. However lasers involving many modes require statistical approaches that treat them as off-equilibrium systems whose stationary regime is brought about by a constant pumping that causes the system to behave as if in equilibrium with a thermal bath: the pumping rate. This allows to liken modes to a liquid and permits to draw phase diagrams of the lasing function^13^. Typically these systems require a mechanism, like disorder in RLs, that establishes the loss channel.

When a large number of modes are involved in the system emission and an interaction between them is considered, it is advantageous to treat the system as a *spin glass*^14^ in the sense that any actual state is composed of a large number of interacting spins that can fluctuate adopting random values to find the equilibrium state. This problem was solved through the use of the *replica trick* for the calculation of free energy^15^ providing an order parameter that was later on proven to bear a physical meaning^16^ and the phenomenon has ever since been referred to as the replica symmetry breaking. In the search to minimize energy some of the possible configurations are blocked because the spins involved cannot comply with the random distribution of couplings in the system (frustration). This theory has been used to model the functioning of ordered and disordered lasers permitting to draw a phase diagram^13^ and was found to account for the modal behaviour of random lasers^17^. Random lasers base the mode interaction in the fact that a proper cavity is lacking and spatial overlap from unfulfilled orthogonality allows an efficient energy exchange. Many modes can be excited by the pumping pulse some of whose interactions are frustrated so that the system is led to choosing between different but equivalent configurations. The set of the activated mode configurations changes from pulse to pulse. Each of these configurations is a *thermodynamic state* characterized by the set of modes activated and their interactions. In our case, as in the case of RLs, under the exact same conditions the systems ends up in different states but, because the distribution of Parisi overlaps between states (of mode configurations) is the same as between replicas^18^, it is possible to identify the RSB from the statistical analysis of overlaps among states. In this framework the result of successive instances of pumping a lasing system can be viewed as equivalent states (conceived as modes configurations) that may present correlations that depend on the states and with non-trivial statistical distributions. While the states are equivalent their correlations may not be. In fact, replica symmetry breaking was so far observed only in RLs because they provide a collection of light modes whose emissions are equivalent (degenerate) and susceptible to frustration. On the contrary, ordinary lasers usually accumulate too few modes under the gain curve to lead to liquid-like behaviour and well defined orthogonality pre-empting frustration. Although quenched disorder is often the fundamental reason for frustration and RSB, some systems with complicated, though deterministic^19^, interactions that can establish self-induced frustration^20^ were shown within the replica theory to display RSB.

In this work we demonstrate a fluctuating behaviour *at* the threshold region of lasers made from pure liquid dye solution in a cuvette and dye doped DNA films *without* adding any scatterers. The fluctuating behaviour is evident from the direct intensity and full width at half maximum (*FWHM*) observations. We have performed measurements to synchronously collect the energy of the pump pulses and the corresponding emission intensity. Our performed measurements demonstrate that the fluctuations are not due to changes in the pulse energy from shot to shot or thermal effects from the sample. Further, the fluctuations in the threshold region are not only present in liquid state lasers but also in solid state although showing comparatively less marked fluctuating behaviour. We have also tested our laser fluctuation for varying pulse duration (τ_p_) and assessed the impact on fluctuations of spatial (through cuvette thickness) and temporal (through the pumping pulse duration) control. Finally, we carried out statistical analyses to prove that mode coupling/frustration is responsible for the fluctuations in the threshold region. The system is the first to show replica symmetry breaking with no intentional disorder because a lousy cavity ultimately induces frustration. This comes about when the immense number of leaky modes involved experience couplings that are frustrated: coherent oscillation of one mode simultaneously with two other coherently oscillating modes can be impossible. This opens the way to equivalent states with different sets of activated modes in each shot.

Two types of systems were tested: liquid-state dye solution laser and solid state dye film lasers. For the former a dye solution was placed in a cuvette and pumped with ns or ps pulses from Q-switched lasers. The latter consisted of dye in DNA films subjected to the same pumping. Figure 1a shows the main features of lasing as a function of pump energy for the typical liquid state configuration. On pumping the cuvette filled with 1 mg DCM in 1 mL THF at low energy density broad photoluminescence from the sample was observed. The *FWHM* for the photoluminescence spectra is ∼60 nm. However, above a certain energy density, a spectral narrowing was observed, accompanied by a significant increase in intensity. For this sample a clear transition from regular broadband (∼60 nm) to narrow band (∼20 nm) laser like emission was observed on increasing the pump energy which is the accepted as a signature of the lasing regime^21^. In the threshold, however, series of spectra at constant pump energy showed that some of the emission peaks have very high intensity with a *FWHM* ∼20 nm (lasing) and some have low intensity and *FWHM* ∼60 nm (Fig. 1b). The two events of lasing and non lasing can be captured on a screen and are shown in the image Supplementary Fig. S1(a,b) respectively. Only rarely the emission has widths and intensities in between (see Fig. 1c) showing a departure from a monotonous behaviour. Figure 1c shows the intensity maxima for spectra collected over 6000 successive laser shots. The lasing or fluorescence behaviour of the successive spectra was clearly observed from the *FWHM* versus shot number plot (Fig. 1d). Notice that, unlike *FWHM,* recorded intensity is limited by the detector dynamic range and all shots of high intensity simply register as the detector maximum making it look like there are many fewer.

To rule out the occurrence of the blinking due to fluctuations of the pump energy that, owing to the rapid change in slope of the emission intensity near threshold, might cause a similar fluctuation in emission we have measured the shot energy synchronously for each spectrum. The normalized correlation coefficient between the intensity and shot energy was calculated according to the expression:


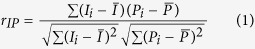


where *I*_*i*_ are the peak maxima and 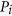 the laser shot energies for the corresponding series of spectra and 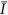 and 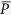 their averages. From Fig. 2a it is evident that the intensity from the sample is well correlated with the shot energy below and above the threshold. But, at or near above the threshold the correlation factor reduces to a value of 0.5 indicating the spectral intensity is decoupled from the shot energy and presents strong fluctuations. Figure 2b shows *FWHM vs*. shot energy for 3000 consecutive shots where we can see that at the threshold (∼0.32 mJ/pulse) the peak widths have two most frequent values (∼60 nm and ∼20 nm) while these values collapse in one or the other away from the threshold region (60 nm below and 20 nm above threshold). This is further detailed in Fig. 2c where a contour plot of the statistical distribution of peak width *P*(*FWHM*) is represented against the pumping energy near the threshold. Shots were grouped in 30 energy segments and the distributions normalized for each segment in the following way. A one hundred-bin histogram of each segment gives *P*_*i*_(*FWHM*) (*i* = 1…30) for thirty energies in the range highlighted Fig. 2a. Each *P*_*i*_ is normalised so that the total probability for each energy adds up to one. These thirty vectors are the columns in the contour plot shown. One can see clearly that at lower energies most of the peaks have widths around 60 nm while for the higher pulse energies this population has diminished and most peaks are narrow with widths of around 20 nm. In the region in between there is a bimodal distribution.

The low intensity-power correlation obtained near threshold is comparable to the degree of time correlation 
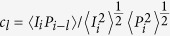
 between successive shots *viz*. lasing intensity, *I*_*i*_, and shot energy, *P*_*i−l*_, as a function of lag (*l*). Notice that shots are separated by one fifth of a second while emitted pulses are in the nanosecond range. Such measurements, as expected, show no correlation between the two except when the lag imposes only two data points in the calculation (*l* = ±3000) making cross-correlation identically equal to 1 merely for mathematical reasons. Away from the threshold region correlation simply attest to the pump lasers stability. (see Supplementary Fig. S2.) This confirms that *r*_IP_ values in the threshold represent fully uncorrelated behaviour. Time delayed intensity–power correlation away from threshold (above or below) shows zero-delay correlation values signalling the fact that in this regime the intensity for each shot is linked to the power causing it.

In order to ensure the findings are not dye dependent other laser dyes like Rhodamine B and also different solvent system (*e.g.* EtOH, H_2_O, DMSO, acetonitrile, ethylene glycol etc.) were tried. In every case we find the same behaviour at the threshold region. The fluctuation behaviour is independent of the solvent-solute interaction, viscosity and boiling point of the solvents. To further ascertain that the fluctuations of the intensity at the threshold are not due to the thermal or other effects from the solution we have made several samples with different concentration of DCM dye in THF so as to place the threshold at different absolute pulse energies. As we increase the concentration the lasing threshold increases drastically as can be seen from the Supplementary Fig. S3a. The required threshold energy increases one order of magnitude from 261 μJ to 2.4 mJ, as we increase the concentration from 0.05 mg/mL to 3.2 mg/mL. This is believed to be due to the inner filter effect^22^ as well as internal quenching. It is also interesting to note that at the highest concentration the samples show fluctuation not only at the threshold but also above threshold due to pump power limitations. Gain is low and the region of ordinary lasing above threshold is pushed too far towards high energy and beyond the limits of the available laser and probably the dye tolerance. However, the intensity fluctuation at the threshold was observed for all the samples. When the threshold is low there is ample margin to pump much above threshold before any sign of saturation shows (see Fig. 1a). If the threshold is pushed higher, when stimulated emission sets in the system is near saturation and the fluctuations cannot be damped.

In order to establish it further, beyond photophysical properties, we have prepared samples of various solutions having a range of boiling point *viz*. THF → EtOH → DMSO → Diethylene Glycol. The solvents have an increasing boiling point starting from 66 °C up to 244 °C. The concentration of DCM in all the cases was kept constant. The intensity fluctuations were present for all the samples. These observations prove that the fluctuations in the intensity of the samples are not due to the thermal effect of the samples and clearly support the hypothesis that the fluctuations are not due to environmental factors but are intrinsic to the system. Next we intended to measure solid state samples to check whether the fluctuating behaviour is also present in that case. We prepare a DNA-CTMA complex doped with DCM dye as previously developed in our laboratory^23^. The sample, in the form of a thin film of several hundred micrometres thickness, was pumped by a stripe formed by the cylindrical lens and the emitted light was collected from the edge. For this particular sample the intensity fluctuations were not as evident from the successive spectra collected at different pump energies. The plot in Supplementary Fig. S4(b) shows thirty consecutive spectra at the highest fluctuation point.

To obtain more insight into the fluctuation from different samples (liquid and solid), it is instructive to analyse the fluctuation coefficient (*f*) defined as the standard deviation (σ_*I*_) of the intensity of light emitted by the sample divided by the mean of the intensities 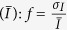 so that we can describe the strength of the fluctuations by examining their statistical distribution. At threshold when fluctuation is strongest the probability distribution is not Gaussian. Instead they follow U-quadratic distribution (see Supplementary Fig. S5.) Fig. 3a shows the statistical analysis of *f* as a function of pump energy for the liquid sample for the solid film. It is evident from the plot that *f* has maximum at the threshold and minima at the regions below and above threshold. This is clearly at odds with related results in RLs where the variance of the emitted intensity scales as the intensity itself^17^. Figure 3b shows the statistical distribution of *FWHM* below (green), at (orange) and above (red) the threshold. In all three cases and despite the difference in ranges data was processed into one hundred bins which makes the histogram bars thinner away from the threshold. Both below and above the distributions clearly resemble a normal distribution. At threshold however the distribution (orange histogram) greatly departs from normal as can be seen in Fig. 3b where *f* takes its maximum value. It is interesting to note that *f* reaches values as large as 152% to be compared with values for the laser that never go above a few. In fact the fluctuations at the threshold are so large that, owing to its limited dynamic range, the detector saturates which impedes to record a good distribution, something that the *FWHM* permits very clearly. The fact that some of these pulses are so much more intense than the average makes the sporadic lasing events directly observable by the naked eye as a red blinking point on a constant green background (that corresponds to the pump laser). Figure 3c shows a similar analysis for the intensity: here the range of the random variable is so wide that separate logarithmic scale plots are needed. Again, while below and above the distributions are ostensibly Gaussian, in the threshold a clear bimodal distribution appears. The threshold region of the solid state laser also shows fluctuations that reach a maximum value of ∼27%, well above the pumping laser pulses.

## Mode coupling

The dynamics of the *single* mode laser field as it interacts with the lasing medium has been examined in the context of non-equilibrium statistical mechanics so that a laser near threshold can find a close parallel in the order/disorder phase transition of a pure fluid vapour-liquid second order phase transitions. The interaction of a molecule’s emission with that of all other molecules is entirely similar to that of a magnetic dipole with its environment in a ferromagnet. This invites to identify the laser electric field as the variable corresponding to the ferromagnetic order parameter and the population inversion as the temperature^24^. Our systems, despite their apparent similarity to ordinary rather than RLs, contain many modes and, owing to low quality of the cavity, we believe that mode coupling is responsible for the intensity fluctuations. Other possible causes like random losses; local change of molecules concentration which might rapidly change the threshold or the optical feedback like chaotic seeding can probably be ruled out by the independence on physical and chemical environments as tested here.

**Figure 4:**
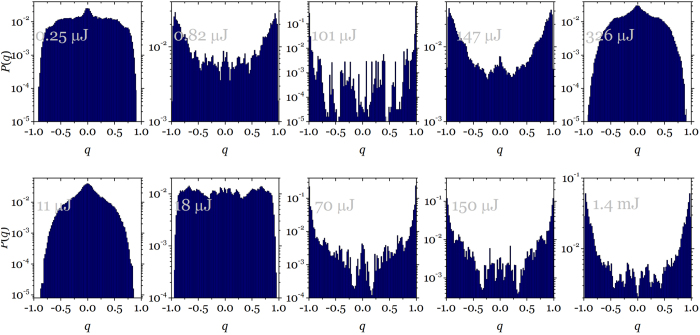
Overlap distribution as a function of pumping. Plot of *P*(*q*) versus *q* for different pumping rates from below threshold to above threshold and at the threshold region for liquid state sample (**upper row**) and below and on threshold for solid state sample (**lower row**). Each panel carries the pump energy as a label and can be identified in Fig. 1a and Fig. 3a. by the larger symbols. All distributions in relative frequency to insure integral equals unity.

In the case of the liquid sample the cuvette cavity acts as a resonator. The longitudinal mode spacing for a regular Fabry-Perot is given by 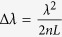. Here *L* is the cavity length and *n* is the refractive index of the medium. The calculated mode spacing, Δλ = 0.068 nm and 0.0135 nm for 2 mm and 10 mm path length cuvette respectively. These values are well below the resolution limit of the spectrometer (0.4 nm). The number of modes per unit volume supported by a cavity can be expressed as 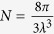; which gives 3.8 × 10^10^ modes per cubic millimetre, for light of 604 nm wavelength. For the excitation of an area of 25 μm^2^ and considering the path length of the cuvette 10 mm ~9.53 × 10^7^ modes are activated along the cylindrical length (π*R*^2^*L*). This immense number of modes sets this system apart from early studies in few modes semiconductor lasers just as the fact that the cavity is regular rather than disordered gives it a novel character at variance with RLs. It is therefore an unlikely environment for ordinary lasers threshold instability and for replica symmetry breaking but the latter is proved by the analysis of shot to shot correlations. To assess the establishment of a regime of replica symmetry breaking induced by coupling between the modes as a function of pump energy we followed a statistical mechanics approach. Just because the distribution of overlaps between mode states is the same as between mode replicas, it is possible to detect the replica symmetry breaking from the statistical analysis of the former^18^, it is possible to detect the replica symmetry breaking from the statistical analysis of the former. For each shot, *α*, we evaluate the intensity fluctuation of the modes (labelled by wavelength, *k*) 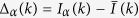 where *I*_α_(*k*) is the intensity of mode *k* for shot *α* and 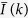 is the average over all shots at mode *k.* The overlap of the spectral fluctuation from shot to shot can be calculated by the correlation between intensity fluctuation of any two shots *α* and *β*:


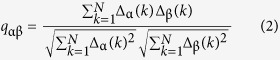


where the sums run over the number of modes (*N*). This analysis is based on the intensity alone, as the phase is hard to obtain, but it has shown its power in revealing the different stages RL presents in terms of replica symmetry breaking^25^. Figure 4 shows the plot of statistical distribution of *P*(*q*) versus *q* calculated for *α, β* = 1, … 500 shots that provide a total 500 × (500 − 1)/2 values of *q*. The plots are in log scale to try to show as much detail as possible in the cases where the distribution presents a large range of probability densities. At very low pulse energies (0.25 μJ in Fig. 4 upper row) the distribution presents a peak centred at *q* = 0 on top of a much weaker background. This same behaviour is found at very high pulse energy (326 μJ in Fig. 4 upper row) and can most possibly be a sign that a regime where full RSB is attained. At pulse energies very near above and below the threshold the distribution presents the signature of one-step plus full RSB with a peak around *q* = 0 and two wings reaching *q* = ±1. In the threshold energy range (101 μJ in Fig. 4 upper row) the distribution is totally different with two strong maxima at *q* = ±1 and a largely depleted region around *q* = 0 pointing to a one-step RSB. In these circumstances where emission consists of broad weak peaks and narrow, intense lasing pulses if the statistical analysis is performed after separating both kinds of spectra, two differing statistics are found: weak peaks obey a replica symmetric zero-centred distribution while intense laser bursts follow a one-step RSB distribution with high density at *q* = 0 (refer to SI Fig. S6).

It is very clear from (Fig. 4) that in the threshold regime (*P* = 101 μJ) the range of small |*q*| is depleted indicating that any two shots have very strongly correlated intensity fluctuations over wavelength. Either upswings in some modes coincide with upswings in others (*q* ~ 1) or upswings coincide with downswings (*q* ~ −1). In other words, in this regime the electromagnetic modes strongly interact and become coupled. On the other hand, if we prepare the system in the region below or above threshold the modes are mostly uncorrelated as revealed by zero peaked distribution.

**Figure 5:**
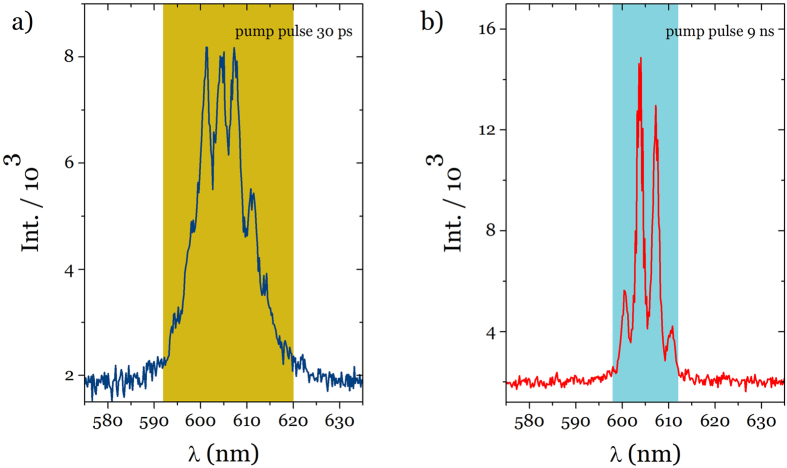
Pumping pulse length. The spectra obtained for a same sample keeping the exact set up conditions. (**a**), for *pico* pumping and (**b**), for *nano* pumping condition. Shaded areas represent the spectral range for two pumping conditions showing that sustained pumping enables mode interaction and laser emission narrowing.

If the fluctuations in the intensity at threshold are mediated by coupling of the modes and frustration, which are activated during the pump pulses, they should respond to the time dependence of the excitation. The coupling is more effective if the pulse duration (τ_p_) is comparable to or much longer than the mode lifetime. The mode lifetime is in the order of hundreds of picoseconds^26^,^27^. The experimental set up (Supplementary Fig. S1) allows nanosecond laser pulses (τ_p_ = 9 ns) and picosecond laser pulses (τ_p_ = 30 ps) to be sent to the sample without disturbing any other part of the set up. Unlike in the case of *nano* pumping, during the *pico* pumping as we sweep the pulse energy through the threshold region, no fluctuations are observed in the intensity emitted. Moreover the effective spectral window over which the lasing peaks are found for ns pulses (14 nm) is narrower as compared to the range generated by picosecond pulses (32 nm) as can be seen in Fig. 5. The time required to establish the coupling between modes makes that in case of *nano* pumping a larger number of modes is activated whereas in the case of *pico* pumping only strongly interacting modes can establish an effective coupling in the duration of the exciting pulse^6^,^28^.

It is also possible to act on the intensity fluctuation through a spatial control. By making the sample thinner and thinner, eventually we decrease the number of modes to a point where there is not sufficient direct (mode to mode) and mediated (through a third mode) coupling between modes so that they can’t couple effectively and subsequently be liable to frustration, to produce a large intensity fluctuation. They can only show a relatively small fluctuating behaviour as was seen from our solid state sample.

In conclusion, we have studied the fluctuating behaviour of the emission spectra of fabricated solid state and solution state lasers. Because of the high gain of the liquid dye solution the coupling between the modes is so effective that replica symmetry breaking becomes a readily observable phenomenon. Although our system has no deliberate disorder, its modes are allowed to exchange energy and interact but some of these interactions are frustrated obliging the system to choose and leading to equivalent but distinct states. The fluctuation can be explained in terms of mode coupling and frustration. This behaviour is completely intrinsic and can be observed for many dyes. We also show how to manage the fluctuation behaviour by temporal and spatial control. It is also interesting to study this behaviour for large fluorescence lifetime samples (e.g. lanthanides) and in presence of scattering media as well as diffusion time. The ease of fabrication and the simplicity of sample preparation may bring a large variety of application such as use in security marker and photonic displays or even in random number generation.

## Methods

### Preparation of liquid dye solution

1 mg DCM dye was dissolved in 1 mL of THF (tetrahydrofuran). A glass cuvette (internal path length 10 mm) was filled with the THF solution of DCM and mounted on the sample stage for studies.

### Preparation of Solid sample

In a typical procedure at first 10 mg DCM dye was dissolved in 20 mL EtOH by sonication. A previously prepared DNA-CTMA complex (100 mg) was dissolved in 5 mL EtOH. The above two solutions were mixed and stirred for thirty minutes. Films of DCM doped DNA-CTMA on a quartz substrate were prepared by dip coating, keeping them overnight in a calibrated oven at temperature 40 °C.

### Optical set up

The 532 nm laser line was cleaned by using appropriate filters. The beam was split into two parts by a pellicle beam splitter, one part was sent to the energy meter and the other part was focused on the sample using a plano-convex lens (*f* = 10 cm) on a DCM dye solution containing cuvette. The ASE/fluorescence was collected using another biconvex lens (*f* = 10 mm), after removing the pump (532 nm) using a 532/1064 nm notch filter and LP 532 nm, the collimated ASE/fluorescence light was focused on an optical fiber (600 μm core) coupled to the Ocean Optics USB2000+/USB4000 spectrometer (Supplementary Fig. S1). The sample was pumped either by a ND:YAG laser (Litron model Nano-T 250-10) providing 20 mJ, 9 ns, 532 nm pulses operating at 10 Hz or 5 Hz repetitions rate or by a ND:YAG laser (EKSPLA model PL2250) providing 30 ps, 20 mJ, 532 nm pulses operating at 10 Hz repetition rate. To measure correlation between pulse and emission the 532 nm laser beam was split using a 45:55 (R:T) pellicle beam splitter. The energy of the each shot was measured using an energy meter (Gentec-Solo-2) coupled with a detector. To ensure a good synchronization the laser frequency was kept at 5 Hz. 3000 spectra were collected for 10 minutes at several shot energy ranging from 0.0-0.9 mJ.

## Additional Information

**How to cite this article**: Basak, S. *et al*. Large fluctuations at the lasing threshold of solid- and liquid-state dye lasers. *Sci. Rep.* **6**, 32134; doi: 10.1038/srep32134 (2016).

## Supplementary Material

Supplementary Information

## Figures and Tables

**Figure 1:**
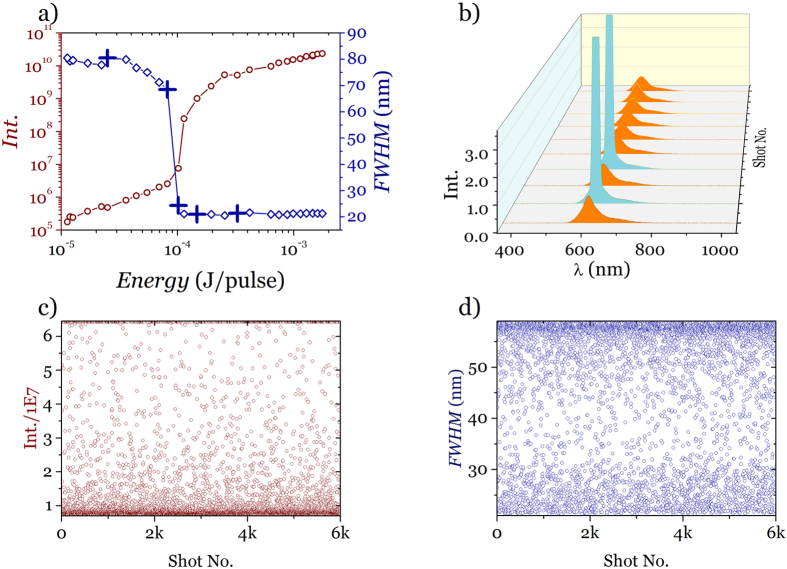
Amplified spontaneous emission and lasing. (**a**) Emission intensity maxima versus excitation energy (532 nm, 9 ns. 10 Hz) red open circles (left axis), and *FWHM* (blue open diamonds) versus pump energy for a dye concentration of 0.04 mg/mL. (**b**) Series of emission spectra obtained from DCM (dye) 1 mg/1 mL THF in successive excitation shots near threshold (pump energy per pulse 1 mJ). (**c**) Emission intensity maxima collected for 6000 shots(pump energy per pulse 1 mJ). (**d**), Corresponding *FWHM* for each spectrum collected in successive 6000 excitation shots. (pump energy per pulse 1 mJ).

**Figure 2:**
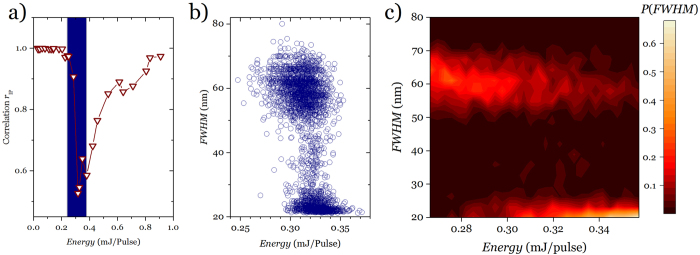
The threshold signatures. (**a**) Normalized emission-pumping correlation coefficient calculated over 3000 single shorts *vs*. the average shot energy; (**b**) the corresponding *FWHM vs.* shot energy at the lowest correlation coefficient and (**c**), probability density of *FWHM* (normalized for each energy) as a function of pulse energy for a dye concentration of 0.04 mg/mL.

**Figure 3:**
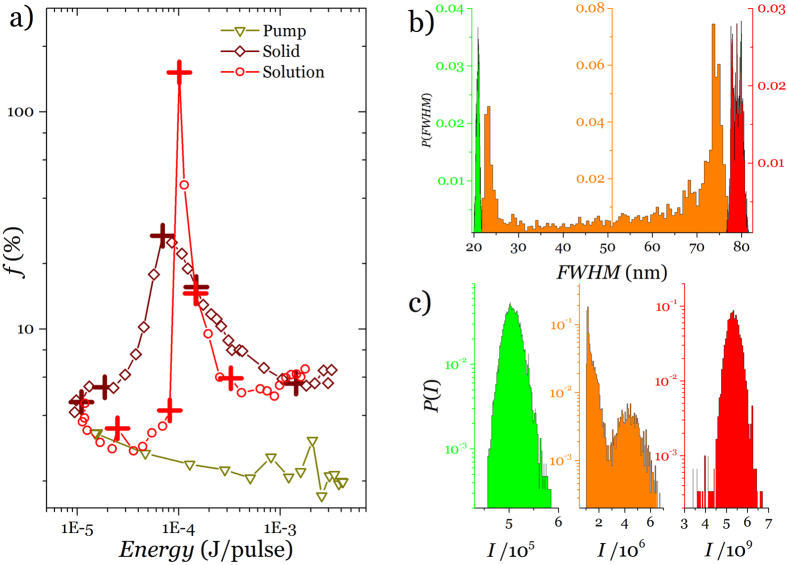
Emission fluctuations at the threshold. (**a**) Fluctuation coefficient (*f*) for DCM dye solution, DCM dye doped DNA-CTMA film and pump. (**b**) Statistical distribution of *FWHM* for dye solution. (**c**) Statistical distribution of intensity. Both distributions expressed as relative frequency so as to insure the integral equals unity.
