# Supplementary material for: Large fluctuations at the lasing threshold of solid- and liquid-state dye lasers
Source: Sci Rep. 2016 Aug 25;6:32134. doi: 10.1038/srep32134 (PMC4997259; doi:10.1038/srep32134)
Supplement: Supplementary Information [file srep32134-s1.pdf]

Large fluctuations at the lasing threshold of solid- and liquid-state dye lasers

Supratim Basak, Alvaro Blanco, Cefe López*

Supplementary Information

S1-Experimental setup (see experimental section for specific details for every element). Briefly, two collinear lasers (ps and ns pulse duration) can be selected for every measurement. Both lasing and no-lasing events can be seen in Figure S1. Images a) and b) captured by the CCD camera show lasing and non-lasing events taken at the same pumping conditions with successive pulses. While a) shows a bright red spot corresponding to an intense narrow emission event, image b) only shows green stray light form the pumping laser.

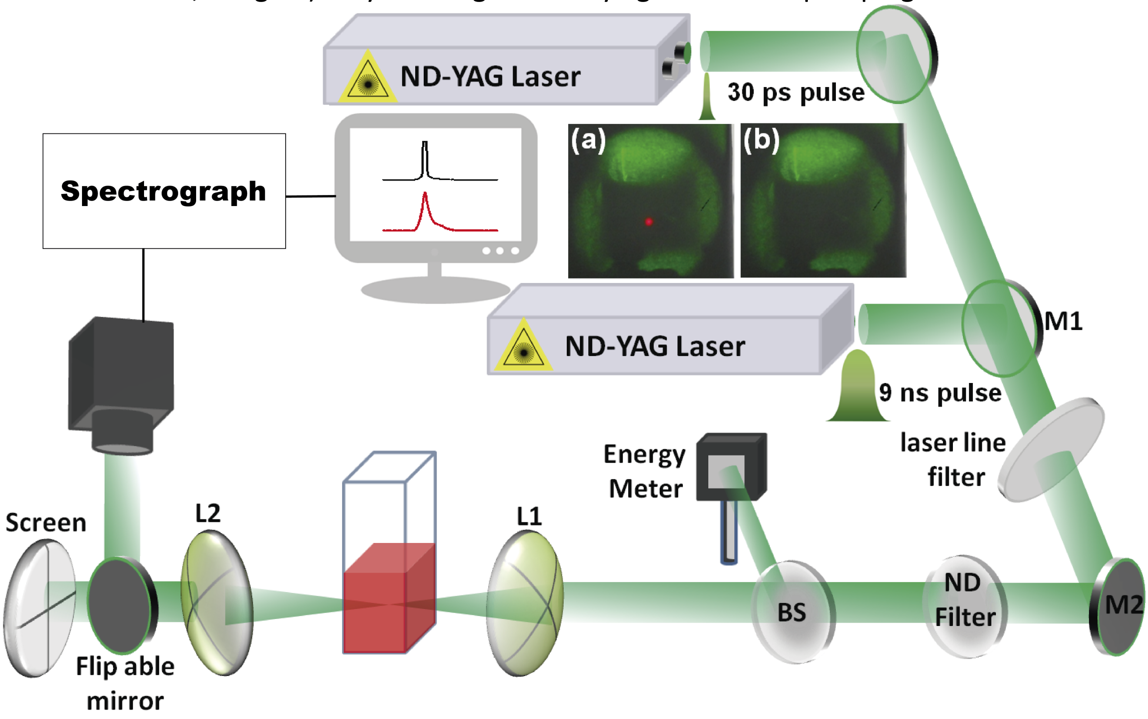

Figure S1 | Experimental set up. The pulse lasers for *nano* (pulse duration=9 ns) or *pico* (pulse duration=30 ps) pumping are sent on the same beamline.

S2-Pumping-Signal Cross Correlation Analysis. The cross correlation $c(l) = \frac{\langle I_i \times P_{i-l} \rangle}{\sqrt{\langle I_i^2 \rangle}\sqrt{\langle P_i^2 \rangle}}$ between intensity and pump energy shows a clear difference between on-threshold and off-threshold as seen in Figure S2. Note that this magnitude represents how the energy of a pulse is related to the lasing produced by preceding pulses.

For on-threshold conditions the overall values are mostly below 0.6 whereas for off-threshold the correlation is almost perfect reaching values very close to unity. The number of data points considered in the calculation N_s-l decreases as the lag (l) approaches N_s the number of shots. In fact when the lag is equal to the number of points only one pair is considered which yields a correlation of one. Off-threshold the high degree of correlation even for non-zero lag only highlights the stability of the systems because if the pump laser had a significant power drift it would appear as a poor cross correlation.

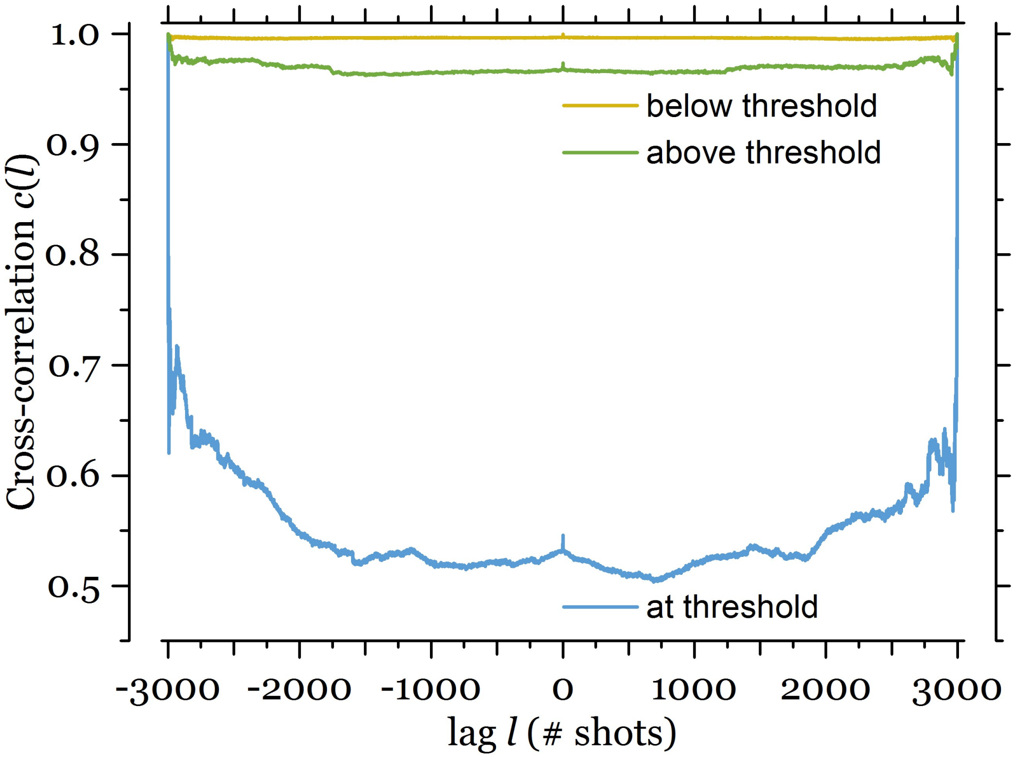

Figure S2 | Normalized cross correlation versus lag position of the two above shown signal.

S3- Dye concentration Next experiment is meant to rule out dye concentration effects other than threshold changes.

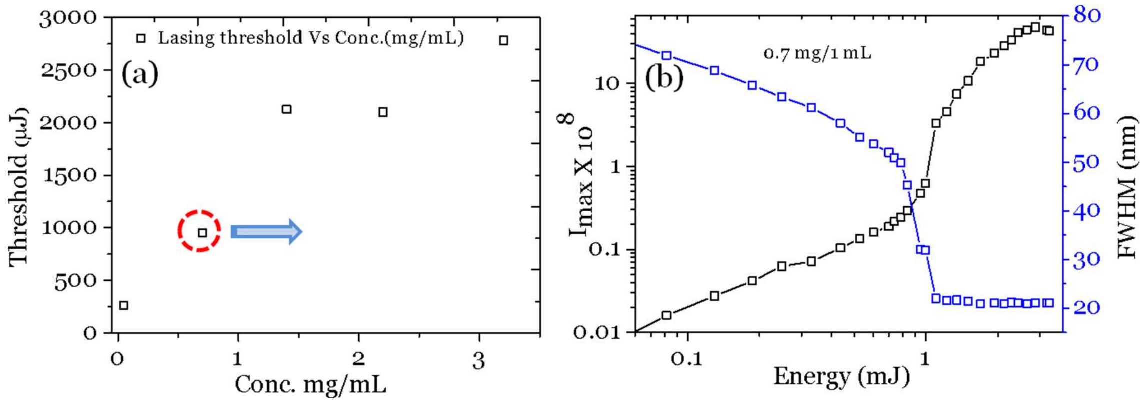

Figure S3 | a, Lasing threshold vs. concentration in mg/mL prepared in THF solvent. **b,** The corresponding I_{max}/FWHM vs. pump energy for the trace surrounded by red dotted circle in **a**.

S4-Solid State Samples Thin solid-state samples were pumped and proved much less fluctuating behaviour, as shown in the example in Figure S4

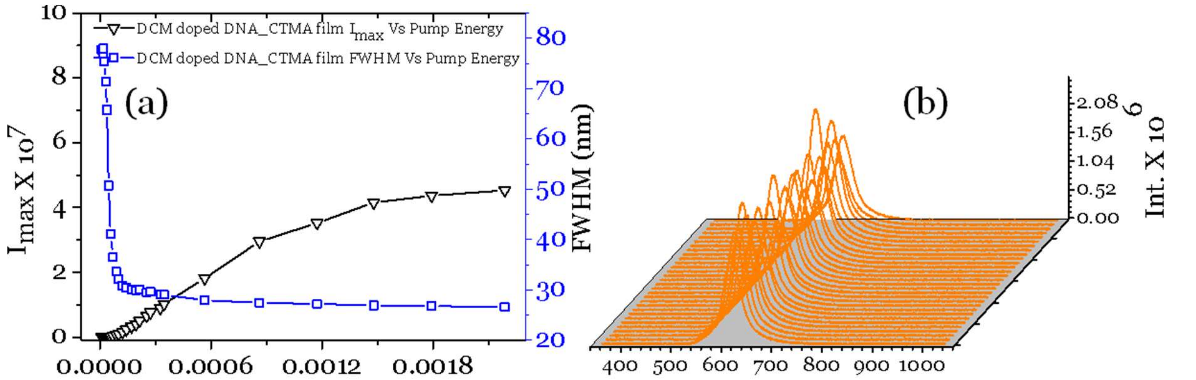

Figure S4 | a, Intensity (black square) and corresponding (FWHM) from the solid state laser as a function of the pump energy. **b,** Series of photoluminescence spectra obtained from the solid state laser in successive excitation shots at threshold.

S5- Fluctuation coefficient at threshold does not follow the Gaussian distribution and U-quadratic is used to fit the results as shown in figure S5.

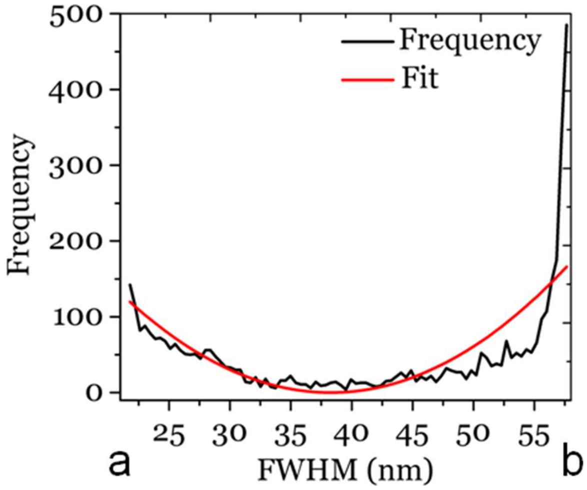

Figure S5 | Fitting of Fig. 3(c) with U-quadratic distribution function

Note: The probability distribution function$F = \alpha(x - \beta)^2$, where $\beta = \frac{a+b}{2}$ and $\alpha = \frac{12}{(b-a)^3}$. *a* and *b* are the point on the *x*-axis corresponds to the maximum frequency. The values obtained from the fit are α = 0.44 and β = 38.

S6-lasing subset analysis. We carried out an analysis of overlap in the threshold by separating the spectra in lasing and non-lasing sets prior to performing the overlap analysis. The results indicate that in the threshold those shots that result in intense, narrow peaks present single-valued overlap distributions concentrated around ±1; corresponding to RS situation like in the third panel in Figure 4 upper row; whereas shots that generate weak, broad emission peaks present the statistics of a RS system like first and fifth panels albeit with a narrower range around zero.

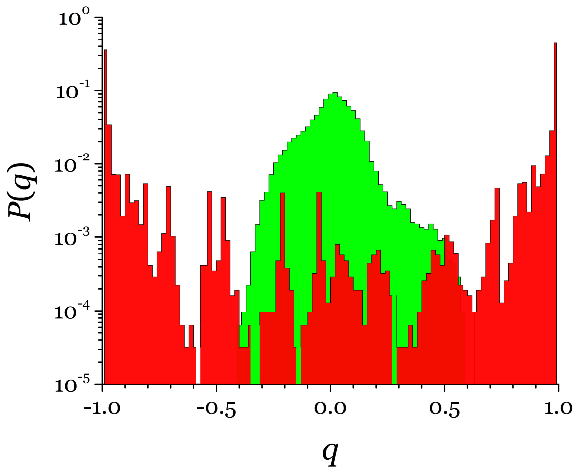

Figure S6 | Statistical distribution of correlations in separate sets of shots. Red (green) histogram represents distribution of overlaps among shots that produce (non-)lasing spectra.
